# General Pavlovian-to-instrumental transfer in humans: Evidence from Bayesian inference

**DOI:** 10.3389/fnbeh.2022.945503

**Published:** 2022-08-16

**Authors:** Luigi A. E. Degni, Daniela Dalbagno, Francesca Starita, Mariagrazia Benassi, Giuseppe di Pellegrino, Sara Garofalo

**Affiliations:** ^1^Center for studies and research in Cognitive Neuroscience (CsrNC), Department of Psychology, University of Bologna, Bologna, Emilia-Romagna, Italy; ^2^Psychometrics and Neuropsychology Lab, Department of Psychology, University of Bologna, Bologna, Emilia-Romagna, Italy

**Keywords:** general Pavlovian-to-instrumental transfer, cue-guided choices, Bayesian statistics in neuroscience, motivation, decision-making

## Abstract

When repeatedly paired with rewarding outcomes (i.e., Pavlovian conditioning), environmental cues may acquire predictive and motivational significance and later enhance instrumental responding for the same (i.e., outcome-specific transfer) or motivationally similar (i.e., general transfer) outcomes. Although outcome-specific and general Pavlovian-to-Instrumental Transfer (PIT) are characterized by different neural substrates and behavioral mechanisms, general transfer has never been studied in isolation from outcome-specific transfer in humans. The first aim of the present study was to test whether the general transfer effect could emerge in isolation and independently of outcome-specific transfer. Our results showed that general transfer can be elicited without the concurrent presence of outcome-specific transfer, supporting the idea that outcome-specific and general transfer can be studied independently of each other. The second aim of the present study was to clarify whether the affordance-like properties of the outcomes can affect the general transfer. In fact, a critical difference in current studies on general transfer concerns the use of cues associated with outcomes for which an action was previously learned (or not) during the instrumental training. This apparently minor difference affects the affordance-like properties of the outcome and may also be transferred to the cue, in turn impacting general transfer. Results revealed a general transfer of the same magnitude regardless of whether cues were associated with reward earned or not during instrumental conditioning. These findings increase the current knowledge on the incentive motivational mechanism behind general transfer, indicating that it is independent of the motor features of the outcome.

## Introduction

Environmental cues (e.g., brand logos) exert a powerful influence on our daily choices. Although neutral in principle, such cues acquire a motivational value through their repeated pairing with a reinforcer (e.g., a chocolate bar), and may bias future choices, driving our reward-seeking behavior (Behrens et al., 2007; Doya, 2008; Watson et al., 2018). For example, a fast-food sign may lead us to that specific fast-food to purchase and eat a hamburger, or it may lead us toward the nearest restaurant to consume food in general.

In the laboratory, cue-guided choices have been investigated using an experimental paradigm called Pavlovian-to-Instrumental Transfer (PIT). The PIT paradigm has been extensively studied in animals (for review, see Dickinson and Balleine, 1994; Holmes et al., 2010; Cartoni et al., 2016). More recently, however, it has become an active area of research in humans as well (Paredes-Olay et al., 2002; for review, see Cartoni et al., 2016; Mahlberg et al., 2021), due to increasing interest in the role of predictive stimuli in guiding actions that are considered maladaptive and where, in general, there is a dysregulation of goal-directed control.

Pavlovian-to-instrumental transfer experiments typically involve three phases: the instrumental conditioning phase, in which participants learn outcome-response associations; the Pavlovian conditioning phase, in which participants learn stimulus-outcome associations; the transfer phase, which tests the ability of the Pavlovian stimulus to affect the instrumental response directed toward the same (outcome-specific transfer) or a similar (general transfer) outcome (Bray et al., 2008; Talmi et al., 2008; Garofalo and di Pellegrino, 2015; Garofalo et al., 2019).

General and outcome-specific transfer effects have most often been accounted for in terms of general or specific influences of Pavlovian cues on instrumental responding, depending on the ability of such stimuli to either enhance responding in general (general transfer), or cue a particular action that produces an outcome that had been previously paired with the stimulus (outcome-specific transfer). There is increasing evidence that each influence on instrumental responding is characterized by a different neural substrate (Corbit and Balleine, 2005, 2011; Prévost et al., 2012; Garofalo et al., 2021), and relies on a separate behavioral mechanism (Holland, 2004; Corbit et al., 2007; Garofalo and Robbins, 2017; Garofalo et al., 2019, 2020).

To date, the findings on human general transfer have been quite heterogeneous. For instance, while some studies report evidence for general transfer using response rate as a dependent variable (Lewis et al., 2013; Quail et al., 2017; Alarcón and Bonardi, 2020b), others failed to observe it (Meemken and Horstmann, 2019; Petrie et al., 2021), or found it only in aversive conditions (Nadler et al., 2011), or using vigor as a dependent variable (Watson et al., 2014; Garofalo and Robbins, 2017). Such heterogeneity in results may be at least partially explained by the lack of studies directly investigating this general transfer effect. Indeed, while most human studies have focused on outcome-specific transfer only (Rosas et al., 2010; Cartoni et al., 2015; Garofalo and di Pellegrino, 2015; Alarcón and Bonardi, 2016, 2020a), a small number has studied both the effects (Nadler et al., 2011; Prévost et al., 2012; Garofalo et al., 2020, 2021), or has investigated a form of transfer in which no distinction could be applied (Talmi et al., 2008; Huys et al., 2011; Geurts et al., 2013; Jeffs and Duka, 2019). To date, the general transfer effect has never been studied in isolation from outcome-specific transfer in humans.

Therefore, the first aim of the present study was to provide evidence about the general transfer effect, by devising a PIT task that did not involve the concurrent study of outcome-specific transfer. Specifically, we examined the ability of Pavlovian cues to enhance instrumental responses that were paired with outcomes (motivationally similar but sensorily) different from that paired with the CS.

The heterogeneity of results in general transfer studies reported above may also be related to several variations in testing procedures. A major difference in the experimental implementation of general transfer concerns the use of cues associated with outcomes for which an action was learned (or not) during the instrumental conditioning phase (Table 1). While many authors operationalized general transfer as the capacity of a Pavlovian cue to bias choice toward an outcome that was never obtained through an instrumental action (Table 1: General PIT_No–action_) (Nadler et al., 2011; Lewis et al., 2013; Watson et al., 2014; Morris et al., 2015; Claes et al., 2016; Lehner et al., 2016; Garofalo and Robbins, 2017; Quail et al., 2017; Meemken and Horstmann, 2019; Alarcón and Bonardi, 2020b; Hinojosa-Aguayo and González, 2020; Krypotos and Engelhard, 2020; Soutschek et al., 2020; van Timmeren et al., 2020; Petrie et al., 2021), others used a different operationalization of general transfer, where the Pavlovian cue predicted an outcome previously earned by a specific instrumental action, which, however, was no longer available in the transfer phase (Prévost et al., 2012; Garofalo et al., 2019, 2020, 2021; Sennwald et al., 2020; Table 1: General PIT_Action_).

**TABLE 1:** Two different operationalizations of general transfer used in the literature.

	General PIT_No–action_	General PIT_Action_
Instrumental	Response_1_ → Outcome_1_ Response_2_ → Outcome_2_	Response_1_ → Outcome_1_ Response_2_ → Outcome_2_ Response_3_ → Outcome_3_
Pavlovian	CS+ → Outcome_3_ CS– → No Outcome	CS+ → Outcome_3_ CS– → No Outcome
Transfer	CS + = Response_1_ + Response_2_ CS– = Response_1_ + Response_2_	CS+ = Response_1_ + Response_2_ CS– = Response_1_ + Response_2_

CS, conditioned stimulus.

Such a seemingly minor methodological difference actually affects the affordance-like properties of the outcome, i.e., its link with a motor program to obtain the outcome itself. According to some theories (Cisek, 2007), for instance, the presence of a response-outcome link may indeed enhance the motivational value of the outcome. Here, we ask whether such affordance-like properties may also be transferred to the CS associated with that outcome during Pavlovian conditioning and, in turn, impact its ability to elicit a general transfer effect. In other words, if we encounter the logo (i.e., a conditioned stimulus) of a food that we never purchased before (i.e., for which there is no motor program available in our past experience), will it prompt us to go get some food, or not?

Therefore, the second aim of the present study was to test whether response-outcome associations affect the general transfer effect. To address this aim, a modified version of the Pavlovian-to-Instrumental Transfer task was developed in order to allow us to directly compare, in each participant, the effect of a Pavlovian conditioned stimulus (CS) paired with an action-associated outcome (CS+_action_), with a CS paired with an outcome that was never associated with an action (CS+_no–action_).

## Materials and methods

### Participants

Thirty-eight volunteers (20 females; mean age = 23.18; sd = 4.97 years; mean education = 15.32; sd = 2.04 years) with no history of neurological or psychiatric diseases were recruited for the study. All participants gave their written informed consent to take part in the experiment. The number of participants was established based on a power analysis conducted on MorePower 6.0 (Campbell and Thompson, 2012), with the following parameters: RM design factors = 1 factor (3 levels); RM effect of interest = 1 factor (3 levels); effect size (η^2^) = 0.12; significance level (Alpha 2-sides) = 0.05; power = 0.8. The effect size was estimated based on the average effect size of all previous studies conducted with a similar task (Garofalo and di Pellegrino, 2015; Garofalo and Robbins, 2017; Garofalo et al., 2019, 2020, 2021).

The study was conducted in accordance with institutional guidelines and the 1964 Declaration of Helsinki and was approved by the Bioethics Committee of the University of Bologna.

### Pavlovian-to-instrumental transfer task

**FIGURE 1:**
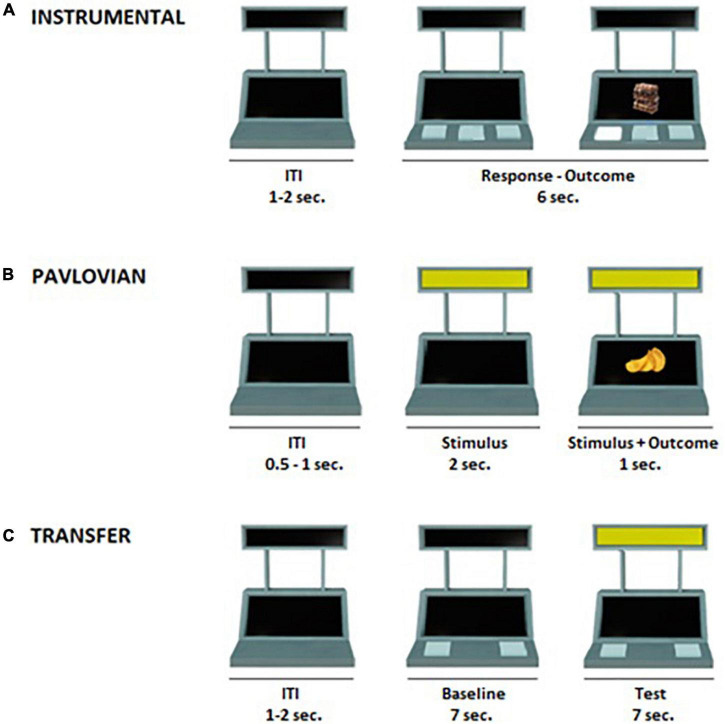
Illustration of the Pavlovian-to-Instrumental Transfer task. **(A)** Instrumental conditioning phase: participants learned the association between three distinct responses and three different food outcomes (e.g., R_1_ → chocolate, R_2_ → crackers, R_3_ → candies). During each trial (6 s), participants were free to press several times the buttons. After each press, the corresponding outcome appeared for 1 second. The inter-trial interval (ITI) lasted 1–2 s. **(B)** Pavlovian conditioning phase: participants learned the association between three distinct colored cues and their respective outcomes. One cue was associated with the outcome corresponding to R_1_ in the Instrumental conditioning phase (CS+_action_; e.g., blue → chocolate), one cue was associated with a new outcome not previously available during the instrumental conditioning phase (CS+_no–action_; e.g., yellow → chips) and a third cue was associated with a non-rewarding outcome (CS–; e.g., red → X). During each trial (3 s) one of the three cues appeared for 2 s, and the outcome was simultaneously presented with the cue during the last second of the trial. During this phase, no response buttons were available. The ITI lasted 0.5–1 s. **(C)** Transfer phase: during each trial (14 s), for the first 7 s (baseline) participants were free to press several times the two buttons as in the instrumental conditioning phase. In the following 7 s, participants were free to press several times the two buttons while the task-irrelevant CS was present. This phase was performed under nominal extinction. The ITI lasted 1–2 s.

The PIT task was structured in three phases, described in detail below, which followed previously validated paradigms (Nadler et al., 2011; Prévost et al., 2012; Garofalo et al., 2021): (1) Instrumental conditioning phase, in which the participant learned a response-outcome association; (2) Pavlovian Conditioning phase, in which the participant learned a conditioned stimulus (CS)-outcome association; (3) Transfer phase, in which the influence of the conditioned stimulus (CS) on the instrumental response was tested. In all task phases, an image of a slot machine was presented in the middle of a computer screen on a white background (Figure 1). The slot machine had two black displays (one on the top and one on the bottom) and three buttons. The task was programmed using OpenSesame3.2 software (Mathôt et al., 2012).

#### Instrumental conditioning phase

In this phase, participants learned the association between the three possible responses (R1, R2, and R3) and their respective rewarding outcomes (O1, O2, and O3) (Table 2). The response consisted of pressing one of three computer keys, corresponding to one of the three buttons of the slot machine (Figure 1A). Each time a computer key was selected, visual feedback was provided such that the corresponding button on the slot machine appeared as illuminated and pressed.

**TABLE 2:** Experimental design of the PIT task.

Instrumental conditioning	Pavlovian conditioning	Transfer
R1 → O1	CS+_action_ →O1	CS+_action_ → R2 + R3
R2 → O2	CS+_no–action_ → O4	CS+_no–action_ → R2 + R3
R3 → O3	CS– → No outcome	CS– → R2 + R3

R, Response; O, Outcome; CS, conditioned stimulus.

For each response, there was a 50% to receive the associated reward. The rewarding outcomes consisted of three food snacks used as separate rewards and presented on the lower display. In non-reinforced trials, a non-rewarding outcome (white “X”) was presented. After each response, the corresponding outcome appeared for 1 s, during which no response was possible. All trials lasted 6 s, during which participants were free to press the three buttons as many times as they wished. During the inter-trial interval (ITI) the slot machine was still visible, but the buttons disappeared, and response options were not available for a jittered duration ranging between 1 and 2 s. Before starting the task, three training trials with no rewards were presented.

The task was structured in a series of blocks to be repeated until a learning criterion was reached. Each block terminated after a total of 24 rewards (8 for each response type) were obtained, for an average duration of about 3 min. At the end of each block, the question “What food did you win by pressing this button?” appeared (one for each response) to test whether all response-outcome associations were correctly established. These blocks were repeated from a minimum of two times to a maximum of eight times. The learning criterion consisted in correctly reporting the response-outcome associations at least two times in a row. If the learning criterion was achieved, the task moved to the following phase. After four wrong answers, the task was aborted.

#### Pavlovian conditioning phase

In this phase, participants learned the association between three colored cues (red, blue, and yellow) serving as conditioned stimuli (CS) shown on the upper display of the slot machine, associated with three separate outcomes, respectively (Figure 1B). The CS+_action_ was paired with the same outcome (O1) associated with response 1 (R1) during the instrumental conditioning phase. The CS+_no–action_ was paired with a food snack serving as new rewarding outcome (O4) not previously available during the instrumental conditioning phase (Table 2), and hence no corresponding response. These two stimuli were randomly rewarded with a 60% reinforcement rate. In the remaining trials, the non-rewarding outcome (“X”) was presented. A third stimulus (CS–) was always paired with the non-rewarding outcome (“X”). Each trial consisted of variable ITI (0.5–1 s), in which the slot machine was “empty” (with no colors or rewards, as in the Pavlovian and instrumental conditioning phase), followed by the appearance of one of the CSs (3 s). The corresponding outcome appeared simultaneously to the CS during the last second. During this phase, no response buttons were represented and hence available.

The task was structured in a series of blocks to be repeated until a learning criterion was reached. Each block consisted of 45 trials (15 for each CS), for an average duration of about 3 min. At the end of each block, the question “What food did you win with this color?” appeared (one for each CS) to test whether all stimulus-outcome associations were correctly established. These blocks were repeated from a minimum of two times to a maximum of eight times. The learning criterion consisted in correctly reporting the stimulus-outcome associations at least two times in a row. If the learning criterion was achieved, the task moved to the following phase. After four wrong answers, the task was aborted.

#### Transfer phase

This phase tested the influence of the Pavlovian conditioned stimuli (CSs) on the instrumental response. Each trial was structured as follows (Figure 1C): first, an empty slot machine (with no colors or rewards) appeared for a variable ITI length (1–2 s); then, two of the buttons previously trained during instrumental conditioning (R2 and R3) appeared for 7 s (baseline); finally, the task-irrelevant CSs (CS+_action_; CS+_no–action_; CS–) appeared for 7 s along with the two response options (test). During both baseline and test, participants were free to press the two buttons as many times as they wished (Table 2). This phase consisted of a total of 36 trials (12 for each CS), for about 10 min.

The whole phase was conducted under extinction, so no rewards were shown. Extinction is a standard procedure for assessing transfer, both in human and animal research, as it allows to test the influence of Pavlovian cues on instrumental responding without the confounding effects of the reward (Colwill and Rescorla, 1988; Bray et al., 2008; Talmi et al., 2008). Specifically, we employed a “nominal extinction” procedure in which participants were instructed that they were still winning but, since the lower display of the slot machine was malfunctioning, they would not be able to see the outcomes (Huys et al., 2011; Quail et al., 2017).

### Procedure

The four rewards were tailored to each participant. Upon recruitment, participants rated the subjective liking of a set of 21 different food items (10 savory foods and 11 sweet foods). For each participant, the experimenter selected four highly and equally valued foods on a 5-point Likert scale ranging from 0 (Not at all) to 5 (Very much). These foods were later used as rewards for the experiment using the same images.

Participants were asked to refrain from eating for 3 h prior to the experiment. Before starting the experimental session, a new liking and wanting 9-point Likert scale, ranging from 0 (not at all) to 9 (very much), were presented for the four foods previously detected, to ensure comparable values between the four rewards. Specifically, we showed the picture of each food and asked to participants the following questions: “How much do you usually like to eat it?” and “How much would you like to eat it now?”, respectively for investigating general liking and current wanting of the rewards. If the participant expressed a preference for one reward over the others, such reward would be substituted with a comparable one. Participants were also asked to rate their current level of hunger.

The experimental session lasted about 45 min and the participant could rest between the phases to prevent fatigue and loss of attention. After providing informed consent, participants were comfortably seated in a silent room and their position was centered relative to the computer screen at about 60-cm viewing distance. The experimenter placed on the table all the food previously chosen by the participant, to ensure a high level of motivation toward the food throughout the task. Participants were informed that, at the end of the experiment, they would receive an amount of food proportional to the number of food pictures visualized during all tasks. In each phase, participants were required to pay attention to the screen and follow the instructions reported at the beginning of the phase.

### Statistical analysis

Analyses were performed with JASP 0.16 (Love et al., 2019) using a Bayesian inferential approach in order to get robust estimates of parameter values and their credible intervals, quantifying support in favor of the null hypothesis (corresponding to the possibility that action and no-action condition may be comparable), and use a model selection procedure (i.e., Bayesian informative Hypothesis) to compare and contrast a broader range of scientific expectations than the standard null and alternative hypotheses (Hoijtink, 2011; Kruschke, 2021).

For Bayesian analyses of variance (ANOVA), the Bayes Factor (BF_10_) is reported as the probability associated with the alternative hypothesis (H_1_) over the null hypothesis (H_0_), along with its estimated proportional error (err%) (Kruschke, 2021). Bayes factor could be summarized in terms of discrete categories of evidential strength. Following the classification proposed in literature (Lee and Wagenmakers, 2013; Andraszewicz et al., 2015), the BF_10_ can be placed on a continuum from “no evidence” (BF_10_ = 1) to “extreme evidence” (BF_10_ > 100), including “anecdotal evidence” (1 < BF_10_ ≤ 3), “moderate evidence” (3 < BF_10_ ≤ 10), “strong evidence” (10 < BF_10_ ≤ 30), “very strong evidence” (30 < BF_10_ ≤ 100). Data are presented as model-averaged posterior distributions and the uncertainty is expressed by the credible interval around the median. Examination of the data distribution ensured that the assumptions for ANOVA were not violated.

In order to provide an assessment of the robustness of the Bayes factor under different prior specifications, a sensitivity analysis was conducted. Sensitivity analysis shows how sensitive the posterior distribution is to the choice of prior distribution: if the qualitative conclusions do not change across a range of different plausible prior distributions, it means that the analysis is relatively robust (Kruschke, 2021; van Doorn et al., 2021).

Estimation plots were used to further illustrate relevant comparisons between conditions (Cumming, 2014; Ho et al., 2019). The web application available at https://www.estimationstats.com/ was used for this purpose. Estimation plots show individual data points for each condition and the paired difference with 95% bias-corrected accelerated confidence interval (CI) based on 5,000 bootstrap samples. Paired differences across conditions were estimated based on the mean (Δ_mean_). The inference was based on the inspection of the estimated difference across conditions (Δ_mean_) and the precision of such estimate (i.e., length of the CI): intervals including 0 were interpreted as indicative of no evidence of effect; intervals not including 0 were interpreted as indicative of weak, moderate, or strong evidence of effect based on the size of the estimated difference and its precision (the longer the CI, the lower the precision, and the weaker the evidence) (Cumming, 2014; Calin-Jageman and Cumming, 2019).

Bayesian Informative Hypotheses were used to provide a joint evaluation of three alternative models reflecting alternative expectations (Hoijtink, 2011; Kluytmans et al., 2012; Gu et al., 2019; Hoijtink et al., 2019b). Each model expresses a specific hypothesis that can be defined in terms of equality and/or inequality constraints among the parameters. For example, three equal parameters can be represented by an equality constrained hypothesis H_0_: A1 = A2 = A3, and three ordered parameters can be represented by an inequality constrained hypothesis H_1_: A1 > A2 > A3. The analysis can also include the unconstrained hypothesis (H_u_), which is a hypothesis representing all possible sets of relationships between the parameters without constraints. The formulation of a model representing the null hypothesis is not mandatory and, as for any other model, should only be included if meaningful from a scientific point of view. For each hypothesis, the posterior model probability (PMP) is calculated *via* the Bayes theorem and expressed with a value between 0 and 1. This value can be interpreted as the relative amount of support for each hypothesis given the data and the set of competing hypotheses included (the sum of all posterior model probabilities adds up to 1). The model with the highest PMP reflects the best hypothesis, i.e., the hypothesis with the highest relative probability (Béland et al., 2012; Hoijtink, 2011; Kluytmans et al., 2012; Hoijtink et al., 2019a,b). To further support model selection, the PMPs can also be compared *via* Bayes factor to (a) that of the other hypotheses tested, (b) to its complement hypothesis (i.e., a model that contains any set of restrictions between the parameters except the one represented by the hypothesis tested), or (c) to the unconstrained hypothesis (Hu) (Hoijtink, 2011; Hoijtink et al., 2019a,b).

## Results

### Liking and wanting

Participants reported comparable liking (BF_10_ = 0.05; err% = 0.69) and wanting (BF_10_ = 0.05; err% = 0.51) values for the four rewarding outcomes, validating the methodological accuracy in the selection of foods. The descriptive statistics are reported in Table 3.

**TABLE 3:** Descriptive statistics for liking and wanting.

	Liking				Wanting			
	Mean	SD	95% credible interval		Mean	SD	95% credible interval	
			Lower	Upper			Lower	Upper
O1	7.39	1.26	6.98	7.81	6.71	1.83	6.11	7.31
O2	7.24	1.32	6.8	7.67	6.39	1.57	5.88	6.91
O3	7.29	1.27	6.87	7.71	6.71	1.90	6.09	7.34
O4	7.45	1.27	7.03	7.86	6.66	1.79	6.07	7.25

### Instrumental and Pavlovian conditioning phases

During the instrumental conditioning phase, all participants successfully achieved the learning criterion. Overall, 97.4% (37 participants) always answered correctly and did not require additional repetitions of the blocks other than the minimum two required, while 2.6% (1 participant) got a question wrong once and had to repeat the blocks for a total three times.

During the Pavlovian conditioning phase, all participants successfully achieved the learning criterion. Overall, 94.7% (36 participants) did not require additional repetitions of the blocks other than the minimum two required, while 5.3% (2 participants) got a question wrong once and had to repeat the blocks for a total of three times.

### Transfer phase

To test the presence of differences among the effect exerted by the three CSs on instrumental responding, we conducted a Bayesian one-way repeated measures Anova with the type of CS as the independent variable (3 levels: CS–, CS+_action_, CS+_no–action_) and the baseline-corrected average number of R2 + R3 in each trial as dependent variable. For baseline correction, the average number of R2 + R3 responses during baseline was subtracted from that performed at test, for each trial. Descriptive statistics for each CS separated for baseline and test are reported in Table 4.

**TABLE 4:** Average response rates for each conditioned stimulus (CS) at baseline and test.

	Baseline				Test			
	Mean	SD	95% Credible Interval		Mean	SD	95% Credible Interval	
			Lower	Upper			Lower	Upper
CS–	3.32	1.07	2.97	3.68	3.21	1.23	2.803	3.61
CS+_action_	3.35	1.06	3	3.70	3.54	1.05	3.199	3.89
CS+_no–action_	3.40	1.11	3.03	3.76	3.58	1.13	3.208	3.95

**FIGURE 2:**
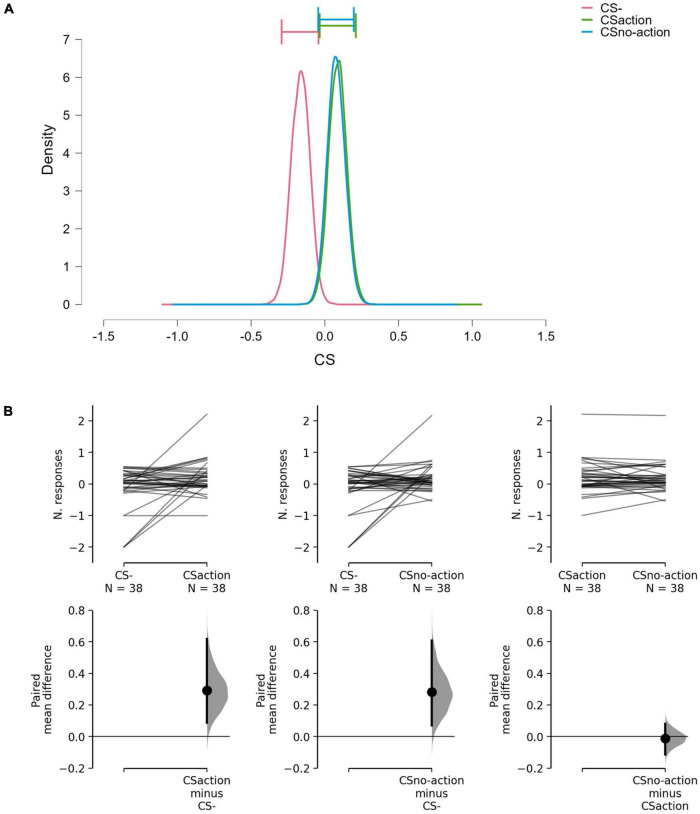
Model-averaged posterior distributions and estimation plots. **(A)** Model-averaged posterior distributions. Horizontal bars show 95% credible intervals around the median. **(B)** Estimation plots show raw data on the upper axes and paired mean difference between CS–, CS+_action_, and CS+_no–action_. On the upper axes, each paired set of observations is connected by a line. On the lower axes, 95% confidence intervals are indicated by vertical error bars, and mean differences, plotted as a bootstrap sampling distribution (5,000 samples), are depicted as dots. Data show increased response rate for CS+_action_ and CS+no-action, compared to CS–, but no evidence of difference between CS+_action_ and CS+_no–action_.

Results showed differences between the CSs (BF_10_ = 3.16; err% = 0.79), suggesting that the alternative hypothesis (H_1_: CS+_action_ ≠ CS+_no–action_ ≠ CS–) predicts the observed data 3.16 times better (moderate evidence) than the null hypothesis (H_0_: CS+_action_ = CS+_no–action_ = CS–). The model-averaged posterior distributions (Figure 2A) show a clear separation between CS– and both CS+_action_ and CS+_no–action_. Estimation plots (Figure 2B) confirmed the presence of increased response rate for both CS+_action_ (Δmean = 0.29, 95% CI [0.09 0.62]) and CS+_no–action_ (Δ_mean_ = 0.28, 95% CI [0.07, 0.61]) as compared to the CS–, and no evidence of differences between CS+_action_ and CS+_no–action_ (Δ_mean_ = –0.01, 95% CI [–0.11, 0.08]).

**FIGURE 3:**
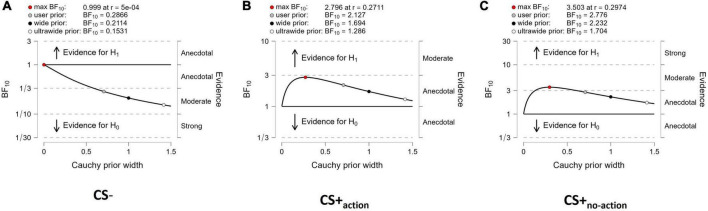
Sensitivity analysis. The Bayes factors are calculated over a range of prior width values from 0 to 1.5. The analysis provides BF10 values over a selection of four prior widths (max: maximum attainable Bayes factor, user: user-specified prior, wide: width of 1, and ultrawide: 1.4), for CS- **(A)**, CS+_action_ **(B)** and CS+_no–action_ **(C)**. The labels on the right of each panel show the robustness of the evidence for the alternative or null hypothesis.

Sensitivity analyses (Figure 3) showed that both evidence for CS+_action_ and CS+_no–action_ (Figures 3B,C), and evidence for the null hypothesis (CS = 0) for CS– (Figure 3A), were relatively stable across a wide range of prior distributions, supporting the robustness of the analysis.

Bayesian Informative Hypotheses were used to clarify whether CS+_action_ and CS+_no–action_ exert a different influence over response rate, relative to CS–. More specifically, we formulated three hypotheses about response rate, which were tested against each other. The first hypothesis posited that the CS+_action_ exerted a stronger influence over response rates than CS+_no–action_:

H:1CS+>actionCS+no-action

The second hypothesis posited that the CS+_no–action_ exerted a stronger influence over response rates than CS+_action_:

H:2CS+>no-actionCS+action

The third hypothesis posited that CS+_action_ and CS+_no–action_ exerted an equally stronger influence over response rates than CS–:

H:3(CS+=actionCS+)no-action>CS-

**FIGURE 4:**
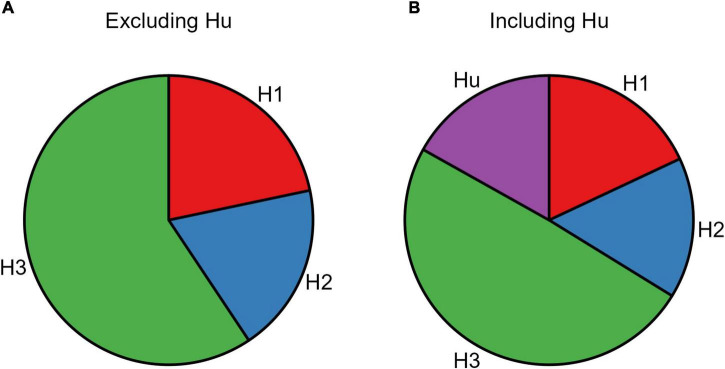
Pie chart of the posterior model probabilities. The two pie charts represent the posterior model probabilities associated with the three models (H1, H2, and H3), when excluding **(A)** or including **(B)** the unconstrained hypothesis (Hu).

The resulting posterior model probabilities showed that H_3_ presented the highest relative probability, thus indicating this as the strongest hypothesis both when excluding (PMPa in Table 5 and Figure 4A) or including (PMPb in Table 5 and Figure 4B) the unconstrained hypothesis (H_u_). In line with this, H3 also presented the highest Bayes Factor computed relative to its complement hypothesis (BFc in Table 5) and to the unconstrained hypothesis (BFu in Table 5). Overall, these analyses confirmed that CS+_action_ and CS+_no–action_ conditions exert a similar influence on response rates.

**TABLE 5:** Bayesian informative hypothesis.

	BF.u	BF.c	PMP a	PMP b
H1	1.07	1.15	0.22	0.18
H2	0.93	0.87	0.19	0.16
H3	2.92	120.87	0.59	0.49
Hu				0.17

The table represents the results from the comparison of the three models (H_1_, H_2_, and H_3_) via Bayesian Informative Hypothesis.

BFu, Bayes Factors of the hypothesis in the row vs. the unconstrained hypothesis and complement hypothesis; BFc, Bayes Factors of the hypothesis in the row vs. the complement hypothesis; PMPa, posterior model probability excluding the unconstrained hypothesis; PMPb, posterior model probability including the unconstrained hypothesis.

## Discussion

The first aim of the present study was to test the ability of Pavlovian cues to enhance instrumental responses that were paired with outcomes (motivationally similar but sensorially) different from those paired with the CS. In particular, we aimed to test whether general transfer could emerge in isolation and independently of outcome-specific transfer, as studying general transfer in isolation may be crucial to disentangle the nature of the two transfer effects (Cartoni et al., 2016; Mahlberg et al., 2021). Indeed, Pavlovian cues can exert general motivational effects on behavior by increasing the likelihood of an instrumental response even when cue and response were previously associated with (motivationally similar but sensorially) different outcomes (e.g., Corbit and Balleine, 2005, 2011). This general (Pavlovian-to-instrumental) transfer effect can be differentiated from outcome-specific transfer in which a Pavlovian cue can increase the likelihood of a response associated with the same outcome as that signaled by the cue. Our results show, for the first time in humans, that general transfer can be elicited and therefore studied without the concurrent presence of outcome-specific transfer. Specifically, we found that the presence of cues previously associated with a rewarding outcome (CS+) increased the number of responses as compared to a cue that had been never associated with a reward (CS–).

It is well-established in literature that general transfer reflects a motivational process (Holland, 2004; Corbit and Janak, 2007). During a decision, when a Pavlovian cue cannot drive your choice toward one of two outcomes, it enhances the general vigor of your action, due to the motivational commonalities between the outcomes currently presented and the outcome that was previously associated with that cue (Dickinson and Dawson, 1987; Dickinson and Balleine, 1990). In other words, a representation of the outcome based on its motivational/affective value (value-based representation) is generated and leads to an association with the CS (independently from its sensory-specific characteristics). It allows to increase the general motivation toward similar outcomes, producing general transfer (Balleine, 1994; Dickinson and Balleine, 2002; Holland, 2004). Conversely, the nature of outcome-specific transfer is more debated. It seems to be mediated by a representation of the outcome based on its sensory-specific features (sensory-based representation), but also the involvement of motivational factors has been hypothesized in the emergence of that effect (Hinojosa-Aguayo and González, 2020). Specifically, together with the sensory-based representation of the outcome, also the value-based representation (Sommer et al., 2022) and the perceived outcome availability (Seabrooke et al., 2019) might drive outcome-specific transfer. Moreover, outcome-specific transfer has been reported to selectively require high-level cognitive abilities, such as working memory (Garofalo et al., 2019), and supraliminal (vs. subliminal) presentation of the reward-associated cues (Garofalo et al., 2020), as well as the involvement of the lateral prefrontal cortex (Garofalo et al., 2021). Within this supposedly hierarchical structure of cue-guided choices, which implies a continuum between low to high cognitive processes, studying general and outcome-specific transfer simultaneously does not allow to establish that the observed general transfer effect is due solely to motivational processes, as it may be influenced by higher cognitive strategies required for outcome-specific transfer, thus creating a possible confound.

Moreover, studying each effect in isolation can inform clinical practice by helping to understand which mechanism is at play in the maladaptive behavior and should, thus, be tackled. In line with this, several studies suggest that an alteration of general transfer contributes to relapse in maladaptive behaviors (for a review, see Doñamayor et al., 2021) like drug addiction (Corbit and Janak, 2007) and alcohol use disorder (Sommer et al., 2017, 2020). The selective involvement of general transfer in maladaptive cue-guided choice suggests that treatments should focus on modifying the motivational aspects of the outcomes involved in the maladaptive conduct. This hypothesis finds preliminary evidence in a study by Schad et al. (2019), which found that, in detoxified patients with alcohol use disorder, alcohol-related outcomes may acquire an aversive value and induce an inhibitory effect, reducing the general transfer effect and the probability of relapse. These results can be interpreted as a reduction of the general transfer due to a natural change in the motivational value of the outcome.

The second aim of the present study was to clarify whether response-outcome associations can affect the general transfer effect. More specifically, we aimed to contrast the effect of a Pavlovian conditioned stimulus paired with an action-associated outcome (CS+_action_), with a CS paired with an outcome that was never associated with an action (CS+_no–action_). In other words, we tested whether manipulating the affordance-like properties of two outcomes (one response-paired and one not) and in turn those of the two associated stimuli (CS+_action_ and CS+_no–action_, respectively), affected general transfer. Our results indicated that general transfer is found regardless of the affordance properties of the CS. Indeed, the number of responses to the CS+_action_ was comparable to those of the CS+_no–action_.

Together our results expand the current major theoretical accounts of the transfer effect, which state that general transfer is independent from the sensory-specific characteristics of the outcome, by adding that it is also independent from its motor-related characteristics. Indeed, the presence (or absence) of affordance-like information associated with the CS modulated neither the probability nor strength of the general transfer effect. This indicated that, at least behaviorally, previous experience with the action or the motor program that leads to the desired outcome does not impact the effect that an environmental cue can have on choice (Starita et al., 2022). To answer our initial research question, even seeing the logo of food that we never purchased before can drive us to get some food.

Crucially, these observations may not apply to outcome-specific transfer, which may be more sensitive to the motor properties of an outcome and thus possibly transferred to the associated CS. Furthermore, such absence or difference at the behavioral level may or may not be reflected at the neural level. Future studies may try to clarify that.

In conclusion, the present findings constitute the first evidence that general transfer can emerge independently of outcome-specific transfer in humans, supporting the idea that the incentive motivational mechanism behind general PIT is independent of the motor features of the outcome.

## Data availability statement

The datasets presented in this study can be found in online repositories. The names of the repository/repositories and accession number(s) can be found below: https://osf.io/57uz6/.

## Ethics statement

The studies involving human participants were reviewed and approved by the Bioethics Committee of the University of Bologna. The patients/participants provided their written informed consent to participate in this study.

## Author contributions

SG, LAED, FS, and GdP conceived and developed the main idea and study design. LAED and DD carried out testing and data collection. LAED and DD performed the analysis under the supervision of SG and GdP. LAED wrote the main manuscript text in collaboration and according to the critical revisions of DD, SG, GdP, FS, and MB. All authors read and approved the final version of the manuscript.
